# Determining the order of resistance genes against *Stagonospora nodorum* blotch, Fusarium head blight and stem rust on wheat chromosome arm 3BS

**DOI:** 10.1186/s13104-016-1859-z

**Published:** 2016-02-02

**Authors:** Rima Thapa, Gina Brown-Guedira, Herbert W. Ohm, Maria Mateos-Hernandez, Kiersten A. Wise, Stephen B. Goodwin

**Affiliations:** Department of Agronomy, Purdue University, 915 W. State Street, West Lafayette, IN 47907-2054 USA; USDA-ARS Plant Science Research, North Carolina State University, Raleigh, NC 27695-7620 USA; Monsanto Company, 1982 State Route 46, Stonington, IL 62567 USA; Department of Botany and Plant Pathology, Purdue University, 915 W. State Street, West Lafayette, IN 47907-2054 USA; USDA-ARS, Department of Botany and Plant Pathology, Purdue University, 915 West State Street, West Lafayette, IN 47907-2054 USA

## Abstract

**Background:**

*Stagonospora nodorum* blotch (SNB), Fusarium head blight (FHB) and stem rust (SR), caused by the fungi *Parastagonospora* (synonym *Stagonospora*) *nodorum, Fusarium graminearum* and *Puccinia graminis*, respectively, significantly reduce yield and quality of wheat. Three resistance factors, *QSng.sfr*-*3BS*, *Fhb1* and *Sr2*, conferring resistance, respectively, to SNB, FHB and SR, each from a unique donor line, were mapped previously to the short arm of wheat chromosome 3B. Based on published reports, our hypothesis was that *Sr2* is the most distal, *Fhb1* the most proximal and *QSng.sfr*-*3BS* is in between *Sr2* and *Fhb1* on wheat chromosome arm 3BS.

**Results:**

To test this hypothesis, 1600 F_2_ plants from crosses between parental lines Arina, Alsen and Ocoroni86, conferring resistance genes *QSng.sfr*-*3BS*, *Fhb1* and *Sr2,* respectively, were genotyped and phenotyped for SNB along with the parental lines. Five closely linked single-nucleotide polymorphism (SNP) markers were used to make the genetic map and determine the gene order.

**Conclusions:**

The results indicate that *QSng.sfr*-*3BS* is located between the other two resistance genes on chromosome 3BS. Knowing the positional order of these resistance genes will aid in developing a wheat line with all three genes in coupling, which has the potential to provide broad-spectrum resistance preventing grain yield and quality losses.

## Background

Fusarium head blight (FHB), stem rust (SR), and *Stagonospora nodorum* blotch (SNB), incited by the fungi *Fusarium graminearum*, *Puccinia graminis* f. sp. *tritici*, and *Parastagonospora* (synonyms *Phaeosphaeria*, *Stagonospora*) *nodorum*, respectively, cause yield losses up to 50 % or more in wheat (*Triticum aestivum*) when favorable environmental conditions enable severe epidemics [1–4]. FHB reduces test weight and lowers the market grade of wheat grain. Symptoms of FHB include bleaching and premature death of cereal spikelets; signs on glumes include white or pink fungal growth that becomes visible under humid conditions [5]. The causal fungus produces various toxins including deoxynivalenol (DON), commonly called vomitoxin, because it induces symptoms of nausea and vomiting in mammals [6, 7]. Grain that is heavily contaminated with DON cannot be sold for food or feed, leading to a complete loss of the crop after harvest. The primary inoculum for the disease comes from airborne sexual (ascospores) or asexual (macroconidia) spores produced on debris from previous crops including stalks and stubble of maize, wheat and other cereals [8]. In addition, insects such as mites and thrips [9] and wind and rain splash [10] can transport inoculum from infected crop residue to the anthers extruded from wheat flowers; anthers are the most susceptible tissue and the primary site of most initial infections [11]. Outbreaks of FHB have increased during the past 20 years making it currently one of the most challenging fungal diseases of wheat [12].

FHB resistance is polygenic [13], and symptom expression is highly influenced by the environment. Two major types of resistance, I and II, have been identified that can be effective against FHB in wheat [14]. Type I resistance reduces initial infection and is assessed as disease incidence. Type II resistance reduces fungal spread within wheat spikes and is assessed as the number of infected spikelets within a spike after inoculation into a single floret. *Fhb1* (also referred to as *QFhs.ndsu*-*3BS*) is a major gene for resistance against FHB that is located in the distal region of wheat chromosome arm 3BS [15]. Alsen, a spring wheat cultivar released recently by North Dakota State University, is a source of type II resistance provided by *Fhb1* [16]. Molecular marker UMN10 is closely linked to *Fhb1* and can be used for marker-assisted selection (MAS) for FHB resistance [16].

Stem rust is another important fungal disease wherever wheat is grown. Signs of SR appear first on the stem as diamond-shaped pustules, which develop into larger lesions on the plant epidermis [5]. Each pustule contains urediniospores that give infected plants a rust-red color. When plants mature, black teliospores are produced in the pustules, hence the name black stem rust. Many epidemics of SR during the past 80 years have reduced wheat yields by 50 % in the Great Plains of the United States [1–3]. SR is managed typically with host resistance, but the pathogen has shown a remarkable ability to overcome introduced resistance genes. The resulting “boom and bust” cycles have led to periodic epidemics when resistance breaks down [17], emphasizing the need for a focus on SR resistance in wheat breeding programs. Interest in SR has increased since 1998 with the discovery of race TTKSK (also known as Ug99) in eastern Africa [18]. This race is concerning because it overcomes *Sr31*, a resistance gene introduced into wheat from rye [18]. This gene previously provided resistance to 90 % of the world’s wheat crop. Race TTKSK is currently spreading across Africa, Asia and the Middle East and poses a major threat to global wheat production [17].

SR resistance gene *Sr2* provides broad-spectrum protection against this disease [19]. This gene was transferred from tetraploid emmer wheat (*Triticum dicoccum*) into the susceptible bread wheat cultivar ‘Marquis’ during the 1920s [20]. *Sr2* has a recessive inheritance and provides adult-plant resistance [21, 22] against all known pathotypes of stem rust including race TTKSK (Ug99) [18]. However, it only confers partial resistance and does not provide sufficient protection under heavy disease pressure [23]. Selection for *Sr2* in the field is difficult for plant breeders due to its recessive inheritance and relatively weak phenotype [24]. However, pseudo black chaff, a physiological trait that causes pigmentation of stems and/or glumes, is genetically linked and can provide a surrogate for direct selection on *Sr2* [25].

SNB is another major yield-reducing foliar and glume disease of wheat. SNB has increased in importance worldwide following the introduction and widespread production of dwarf and semi-dwarf wheat lines [26], and by growing wheat lines that are susceptible to toxins produced by the SNB pathogen [27, 28]. In addition, recent adoption of cultural practices such as minimum-tillage agriculture [29–31] and increased use of high-nitrogen fertilizers have increased the impact of this disease. The diagnostic symptoms of the leaf-blotch phase of the disease are chlorotic lesions on the lower leaves that initially are red-brown in color with a yellow halo and eventually develop into tan, lens-shaped lesions containing dark specks of pycnidia producing the asexual pycnidiospores [32]. The glume-blotch phase of the disease occurs later when heads and grains are infected by conidia released from pycnidia and spread by rain-splash dispersal [32].

Several quantitative trait loci (QTL) for resistance to SNB have been identified that provide partial resistance against the disease in wheat [33–38]. SNB resistance gene *QSng.sfr*-*3BS* is present in parental line Arina and has been mapped to wheat chromosome arm 3BS [37]. Arina is a Swiss winter wheat cultivar with excellent resistance to SNB that has been planted to more than 40 % of the Swiss wheat acreage since 1985 [39]. However, the map location of *QSng.sfr*-*3BS* relative to those for *Fhb1* and *Sr2* is not known definitively and all three genes currently are in different genetic backgrounds, complicating their use in wheat-improvement programs.

The objective of this analysis was to determine the order of resistance genes *Fhb1*, *Sr2* and *QSng.sfr*-*3BS* to test the hypothesis that *QSng.sfr*-*3BS* occurs between the other two genes on wheat chromosome arm 3BS, with the long-term goal to obtain all three genes in coupling. The first objective was achieved by crossing three parental lines with resistance to *Fhb1*, *Sr2* or *QSng.sfr*-*3BS* to combine the resistance genes into a single background. A segregating F_2_ population of 1600 plants was genotyped with SNP markers to validate the presence of the resistance genes and to determine their order. The entire F_2_ progeny set also was phenotyped for resistance to SNB to test the associations between disease resistance and molecular markers.

## Methods

### Analysis of previously published linkage maps

Resistance genes and/or QTL for various qualitative and quantitative traits located in the 3BS region of the wheat genome were identified through literature searches. Linkage arrangements and map locations were obtained from previously published sources [23, 40–45] and a probable genetic linkage map was constructed using MergeMap [46], a free software tool for combining data from multiple sources into a single linkage map based on genetic distances from shared markers.

### Mapping population development

The plant populations used in this study were developed through a two-step process involving three parents with different traits (Table 1). The initial cross was made between the FHB-resistant spring wheat cultivar Alsen, containing *Fhb1* (*QFhs.ndsu*-*3BS*) [16], and the SR-resistant spring wheat Ocoroni86, containing *Sr2* [47], during spring of 2011. Approximately 64 F_1_ seeds were planted immediately after harvest in 30 × 60 × 10-cm plastic trays during the fall of 2011 and placed at 2.8 °C for 7 days to break dormancy. Plastic trays were then brought to a greenhouse until the seeds germinated; greenhouse temperature was maintained at approximately 24 °C during the days and 20 °C at night. Following germination, the trays were moved back to the cold room for 2 weeks and then the seedlings were transplanted into 10-cm-diameter pots and placed in a greenhouse. Day length was 8 h for 21 days, 12 h for 14 days, and then 16 h to physiological maturity; temperature was maintained at 24 °C during the days and 20 °C at night. Plants were fertilized with Miracle-Gro (Miracle-Gro Corporation, Marysville, OH) at the rate of 88.72 mL/3.78 L H_2_O/800 plants. The F_1_ plants were allowed to self pollinate and spikes were covered with glycine bags during flowering to avoid cross pollination.

**Table 1:** Summary information of the three wheat cultivars contributing genes for resistance against *Stagonospora nodorum* blotch, Fusarium head blight and stem rust and summary of simple-sequence repeat markers linked to each resistance gene used for genotyping parental wheat lines during the initial analysis of 400 F_2_ progeny (*Xgwm389*) and for all progeny sets (*Xumn10*)

Cultivar	Growth habit	Resistance gene	Genetic control	Linked SSR markers^a^	Fragment sizes	Reference
Arina	Winter	*QSng.sfr*-*3BS*	Polygenic	*Xgwm389*	*134*-*135*	[37]
Alsen	Spring	*Fhb1 or QFhs.ndsu*-*3BS*	Additive	*Xumn10*	*241*-*242*	[16]
Ocoroni86	Spring	*Sr2*	Recessive	*Xgwm389*	*115*-*119*	[47]

^a^The same PCR conditions were utilized for all SSR markers: initial denaturation at 95 °C for 4 min; 30 cycles of 95 °C for 30 s, 60 °C for 30 s, and 72 °C for 30 s; 30 cycles of 95 °C for 30 s, 45 °C for 30 s, and 72 °C for 30 s; followed by a final elongation at 72 °C for 7 min and a hold at 4 °C

Approximately 400 F_2_ seeds in a plastic tray and 20 seeds of winter wheat cultivar Arina in a 15.24-cm pot were planted, dormancy was broken, seedlings were vernalized and transplanted during the spring of 2012 as described above. The F_2_ seeds were vernalized for only 2 weeks and then the seedlings were transplanted into 10-cm-diameter pots and placed in a greenhouse. Day length, temperature and fertilization were as described above. The pot with the Arina seeds was kept at 2.8 °C for 65 days for vernalization as required to stimulate flowering of winter wheat. All 400 F_2_ plants were genotyped with molecular markers linked to *Fhb1* and *Sr2* (Table 1) to identify those progeny that were likely to be homozygous for both resistance genes. Two plants were identified as double homozygotes and were crossed to the SNB-resistant winter wheat cultivar Arina containing *QSng.sfr*-*3BS*.

The F_1_ seeds from this cross were planted, dormancy was broken, seedlings were vernalized for 65 days and transplanted during summer of 2012 as described above. Day length, temperature and fertilization with Miracle-Gro were as described above. The F_1_ plants were allowed to self pollinate and spikes were covered with glycine bags during flowering to avoid cross contamination.

The resulting F_2_ population, its parents and Chinese Spring as a susceptible control were evaluated for SNB resistance in a greenhouse at Purdue University during January of 2013. Seeds of the F_2_ lines (N = 1600) and parents (N = 20/parent) were planted in 67-cm long × 34-cm wide × 6-cm deep Styrofoam seedling transplant trays with 128 cells. One seed was planted per cell to allow the plants to be genotyped before transplanting. Following vernalization at 3 °C for 75 days, seedlings were transplanted into 10-cm-diameter plastic pots, one seedling per pot during March of 2013. Day length was 11 h for 14 days (22 °C day, 20 °C night), which was increased to 14 h for 7 days (28 °C day, 24 °C night), and then to 16 h (28 °C day, 24 °C night) until maturity. Plants were watered as needed and fertilized with Miracle-Gro twice before transplanting and once with 12-12-12 fertilizer after transplanting.

### Disease screening for SNB resistance in a greenhouse

**Fig. 1:**
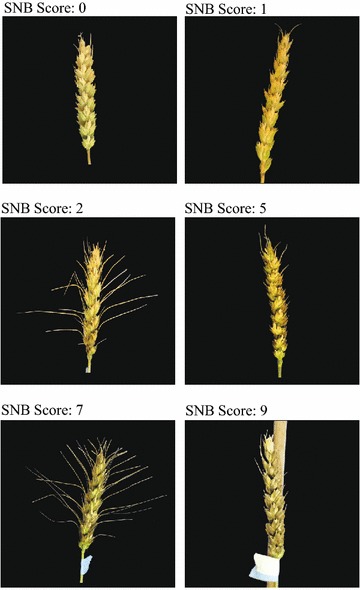
Representative scores of percent diseased glume tissue for the *Parastagonospora* (synonym *Stagonospora*) *nodorum* inoculations. The total scores ranged from 0 (no disease) to 10 (disease covering the whole spike). Representative heads showing scores of 0, 1, 2, 5, 7 and 9 are shown. The degree of awn development also segregated in the cross but did not affect disease scoring

Inoculum of *P. nodorum* was prepared from a field isolate collected from an infected spike of the susceptible wheat cultivar ‘Caldwell’ at Lafayette, IN, USA and cultured on V-8 potato-dextrose agar (PDA) medium following protocols by Friesen et al. in 2012 [48]. The entire 1600 F_2_ progeny and the three parents plus Chinese Spring were phenotyped for resistance to SNB. A vacuum pump sprayer was used for mist inoculation of the primary spike of each plant after spike emergence (Feekes stage 10.3) with approximately 1 mL of inoculum containing 1.6 × 10^6^ conidia/mL of dH_2_O. After inoculation the spikes were covered with a 5 × 10-cm plastic bag for 3 days; the greenhouse benches were flooded at a 2-cm depth during the evenings to increase humidity and enhance SNB disease development. A disease severity score on a single inoculated spike of each plant was assessed using the full range of the 0–10 disease-rating scale (Fig. 1) 21 days after inoculation. Due to wide differences in maturity of the progeny segregating in this winter × spring wheat cross, inoculations were performed in 10 groups as the heads emerged from early to late in the season.

### Genotyping

Genotyping with molecular markers was performed to verify that the crosses were successful and to track the genes of interest. Genomic DNA isolation, PCR and gel electrophoresis were performed as described by Liu et al. in 2013 [49] in parental lines before crossing. SSR markers (Table 1) were used for PCR, based on previously published reports of linkages with the three resistance genes [16, 37, 47] to genotype all crosses and generations except the F_2_ progeny derived from the second cross. A modified touchdown-PCR protocol [50] was used for amplification to increase specificity [51]. All the crosses and generations, except for F_2_ progeny from the second cross, were multiplex genotyped using fluorescent markers. The primers were labeled fluorescently with 6-FAM, NED, VIC or PET. The results were analyzed using SoftGenetics GeneMarker V1.91 (SoftGenetics LLC, State College, PA). Genomic DNA was extracted from F_2_ progeny derived from the second cross using the BioSprint 96 DNA Plant Kit from Qiagen (Germantown, MD). Plants were genotyped using KASPar markers (Table 2) according to the manufacturer’s instructions and end-point genotyping was done using the software KlusterCaller (LGC Genomics, Hoddeson, UK). DNA sequences of markers *Xsnp3BS*-*2*, *Xsnp3BS*-*3*, *Xsnp3BS*-*9* and *Xg01130* were used to design primers for the KASP assays that are amenable to rapid genotyping of large numbers of individuals.

**Table 2:** List of Kompetitive Allele-Specific PCR (KASPar) single-nucleotide polymorphism (SNP) markers and their sequences used to genotype 1600 F_2_ progeny derived from a cross of Arina (*Qsng*-*sfr*-*3BS*) by two F_2_ progeny from a cross between Alsen and Ocoroni86 that were homozygous for the molecular markers linked to the *Fhb1* (Alsen) and *Sr2* (Ocoroni86) resistance genes

Primer name^a^	Sequence	Fluorescent label
*Xsnp3BS*-*2_ALA*	GAAGGTGACCAAGTTCATGCTGGTCTCGGAATTGATTGTAGAAGGTA	FAM
*Xsnp3BS*-*2_ALC*	GAAGGTCGGAGTCAACGGATTGTCTCGGAATTGATTGTAGAAGGTC	VIC
*Xsnp3BS*-*2_C1*	CCAGAGACTACATAKCCTTTTCTTACAAT	
*Xsnp3BS*-*3_ALG*	GAAGGTGACCAAGTTCATGCTTTCAAGTGCTGGCATTGCATCC	FAM
*Xsnp3BS*-*3_ALA*	GAAGGTCGGAGTCAACGGATTCTTTCAAGTGCTGGCATTGCATCT	VIC
*Xsnp3BS*-*3_C1*	GCTTCTTYGCACCTCTAGTGCCAA	
*Xsnp3BS*-*9_ALC*	GAAGGTGACCAAGTTCATGCTTTCAAGAAAGCTTCTGCCAGTCAC	FAM
*Xsnp3BS*-*9_ALG*	GAAGGTCGGAGTCAACGGATTTCAAGAAAGCTTCTGCCAGTCAG	VIC
*Xsnp3BS*-*9_C1*	AAAAGAACAGACACGCCAGGATTCAAAA	
*g01130_AL1*	GAAGGTGACCAAGTTCATGCTATAGCTYCATCTCATTTCCTCCTTG	FAM
*g01130_AL2*	GAAGGTCGGAGTCAACGGATTCATAGCTYCATCTCATTTCCTCCTTA	VIC
*g01130_C1*	TAGAACCAAAGCKTCAAACATTTCTGTGAA	

^a^Primer name indicates the published markers from which the sequences were obtained for design of the KASP assay, with the addition of the allele-specific designation. The common primer is indicated as C1

### Linkage and QTL analyses

The genetic linkage map was constructed using the genotypic data of the F_2_ progeny derived from the cross involving all three resistance genes with the software package JoinMap 3.0 [52]. The Kosambi mapping function was used to calculate the map distances [53]. The orders of the resistance genes within the linkage group were determined via the maximum likelihood (ML) mapping algorithm with an LOD of 10. The genetic map for QTL analysis was constructed using five SNP markers. The phenotypic data for SNB along with genotypic data collected from the F_2_ progeny after the second cross were utilized for QTL analysis. The QTL for SNB resistance was identified via interval mapping in Windows QTL Cartographer version 2.5 [54].

## Results

### Linkage predictions from previously published maps

**Fig. 2:**
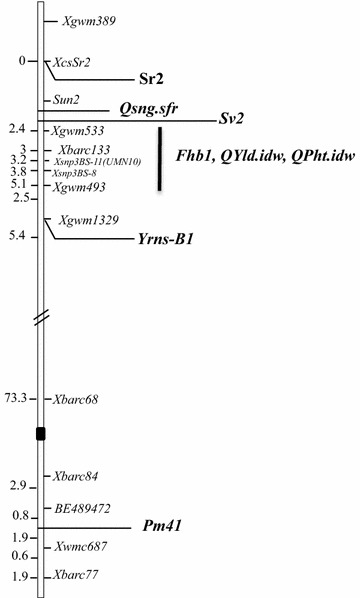
Probable positions of previously mapped genes for resistance to the fungal diseases Fusarium head blight (*Fhb1*), stem rust (*Sr2*) and *Stagonospora nodorum* blotch (*QSng.sfr*-*3BS*) based on published genetic distance from markers shared in common. The *black rectangle* indicates the likely position of the centromere. The approximate distances in centimorgans (cM) between loci on the map are indicated on the *left*. Specific resistance genes and the references (in parentheses) used to generate the probable map are as follows: stem rust, gene *Sr2* [23]; *Stagonospora nodorum* blotch, *QSng.sfr*-*3BS* [45]; leaf rust, *Sv2* [41]; quantitative trait loci (QTL) for yield, *Qyld.idw* and plant height, *QPht.idw* [44]; stripe or yellow rust, *Yrns*-*B1* [42]; Fusarium head blight, *Fhb1* [40]; and powdery mildew, *Pm41* [43]

Analysis of previously published linkage maps suggested that *Sr2* was distal, *Fhb1* was proximal and *QSng.sfr*-*3BS* was in between *Sr2* and *Fhb1* (Fig. 2). Unfortunately, this analysis was not precise enough to indicate whether the SNB resistance gene was closer to *Sr2* or *Fhb1*.

### Mapping populations

To combine the three genes from different parents into a single segregating population, crosses were made initially to identify plants having the *Fhb1* and *Sr2* genes present in the two spring wheat parents linked in coupling. Among 400 F_2_ seedlings tested from the cross Alsen (*Fhb1*) × Ocoroni86 (*Sr2*), two were homozygous for the molecular marker *Xumn10* that is closely linked to *Fhb1* and markers *Xgwm389* and *Xglk683* that flank the *Sr2* resistance gene. The linkage distance between loci *Xumn10* and *Xgwm389* was 16.6 cM. Since the marker analyses indicated that these plants should be homozygous for both resistance genes, they were crossed with Arina (*QSng.sfr*-*3BS*) to develop a population in which all three resistance genes and the linked molecular markers would segregate. A population of F_1_ seeds was created from Arina crossed with each double homozygote and maintained separately. The population with the highest number of F_2_ seeds (approximately 1600) was used to determine the gene order and to make the genetic linkage map.

### SNB evaluation

**Fig. 3:**
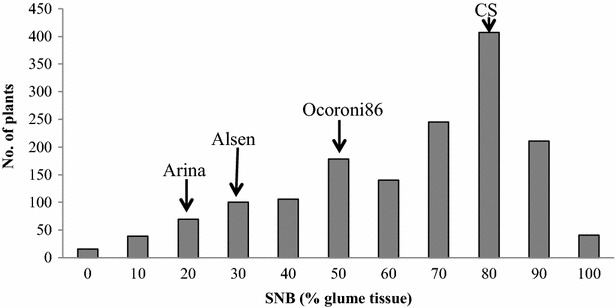
Frequency distribution of the percent diseased glume tissue of 1600 F_2_ progeny derived from a cross between Arina and a doubly homozygous F_2_ progeny from a cross between Alsen and Ocoroni86 inoculated with a field isolate of *Parastagonospora* (synonym *Stagonospora*) *nodorum* in a greenhouse at Purdue University in West Lafayette, IN. CS, the susceptible control Chinese Spring

The histogram of disease scores had a continuous distribution with a skew towards susceptibility (Fig. 3). Transgressive segregation was observed in the population indicating the presence of more than one QTL for resistance.

### Genetic linkage map

In total, 119 simple-sequence repeat (SSR) and SNP loci were screened to identify polymorphic markers in the distal region of chromosome arm 3BS. However, it was difficult to identify markers with different alleles from all three parents. The SSR markers *Xumn10* and *Xgwm389* (Table 1) have been reported as closely linked to *Fhb1* and *Sr2*, respectively, and were used to track the genes of interest after every cross and every generation except for the F_2_ from the second cross, where KASpar markers amenable to high-throughput genotyping were used.

**Fig. 4:**
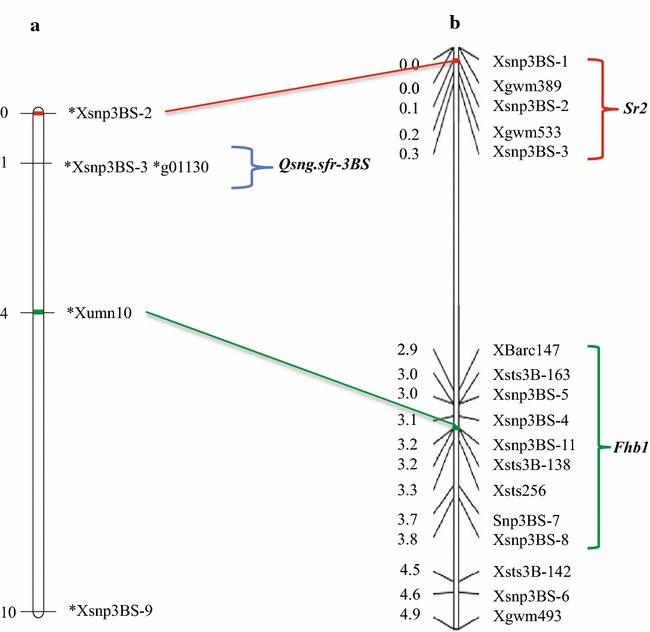
Comparison of our linkage map and a recently published map of the *Fhb1*-*Sr2* region of chromosome arm 3BS. **a** Linkage map of 3BS for markers *Xsnp3BS*-*2*, *Xsnp3BS*-*3*, *g01130*, *Xumn10*, and *Xsnp3BS*-*9* in the F_2_ population of 1600 individuals derived from a cross between Arina and a doubly homozygous F_2_ progeny from a cross between Alsen and Ocoroni86. The map was generated with JoinMap 3.0 (LOD = 9.0). Numbers to the *left* of the *vertical bar* indicate the total distance in centimorgans, *mapped markers* are on the *right*. **b** Adaptation of the Bernardo et al. in 2012 [40] fine linkage map of the 3BS region around *Fhb1*, mapped in a Ning7840/Clark BC_7_F_7_ population. Numbers to the *left* of the *vertical bar* indicate the interval distance in centimorgans (cM), *mapped markers* are on the *right*. The *lines* connecting the figures link the same genetic loci on the two maps. The *green bracket* represents the approximate location of *Fhb1*, the *red bracket* *Sr2* and the *blue bracket* *QSng.sfr*-*3BS*

**Fig. 5:**
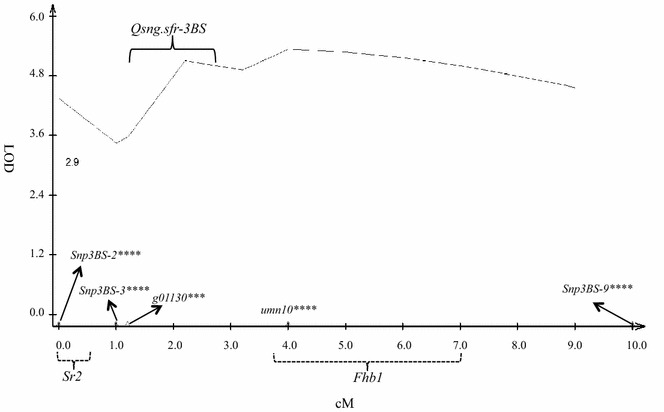
Interval mapping analysis of chromosome 3BS for *Stagonospora nodorum* blotch resistance gene *Qsng.sfr*-*3B* mapped in a 1600-individual F_2_ population derived from a cross between Arina and a doubly homozygous F_2_ progeny from a cross between Alsen and Ocoroni86. LOD values (*y axis*) were determined from a phenotyping experiment of the 1600 progeny for SNB resistance in a greenhouse. Map distances in centiMorgans are indicated along the *x axis*. Marker names are indicated with *arrows* according to their map orders and distances. Probable locations of the resistance QTL are indicated by *solid* or *dashed brackets*, with *dashed* for *Sr2*, *solid* for *Qsng.sfr*-*3B* and *dashed* for *Fhb1*

The F_2_ progeny from the second cross were genotyped with 7 KASPar SNP markers. Five SNP markers were polymorphic among the parents and mapped in a linkage group in the same region as *Fhb1, Sr2* and *QSng.sfr*-*3BS* using the 1600 F_2_ progeny (Fig. 4). Among the five polymorphic SNP loci, *Xumn10* is known to be very closely linked to *Fhb1* and serves as a marker for that resistance gene [40]. The location of this marker places the probable location of *Fhb1* proximal to *Sr2* and *QSng.sfr*-*3BS.* Locus *Xsnp3BS*-*9* was the most proximal of the five loci. Loci *Xsnp3BS*-*3* and *g01130* mapped approximately 1 cM distal to *QSng.sfr*-*3BS* (Fig. 5) based on the first peak associated with the resistant phenotype. *Xsnp3BS*-*2* was most closely associated with the *Sr2* gene in previous research [40] and was mapped to the tip of the 3BS arm and distal to markers associated with *QSng.sfr*-*3BS* and *Fhb1* segregating in our population (Fig. 4). The KASP assay for marker *g01130*, which was associated previously with *QSng.sfr*-*3BS* [55], was located between the markers closely associated with *Sr2* and *Fhb1* in this study.

### QTL analysis for *Stagonospora nodorum* blotch resistance

QTL analysis identified a chromosomal region in the 3BS region of the wheat genome that controls resistance to SNB in the large F_2_ population. When the five markers in our linkage group were tested by the simple linear regression model, all were highly significant by single-marker analysis (Table 3). A highly significant QTL on the distal end of the short arm of chromosome 3B was detected by interval mapping analysis (Fig. 5). The first peak for this QTL was approximately 1 cM proximal to the predictive SNP marker *g01130*, between the markers for *Sr2* and *Fhb1* (Fig. 5). Interval mapping confirmed the significant QTL and showed that the additive and dominance effects for SNB resistance came from the resistant cultivar Arina (Table 4).

**Table 3:** Markers linked to the quantitative trait locus associated with *Stagonospora nodorum* blotch resistance detected by single-marker analysis of 1600 F_2_ progeny derived from a cross of Arina (*QSng.sfr*-*3BS*) by an F_2_ progeny from a cross between Alsen and Ocoroni86 that was homozygous for the molecular markers linked to the *Fhb1* (Alsen) and *Sr2* (Ocoroni86) resistance genes

Marker	Single-marker analysis^a^			
	b0	b1	pr(F)	R^2^ (%)^b^
*Xsnp3BS*-*2*	6.393	−0.362	8.8E-06****	1.33
*Xsnp3BS*-*3*	6.270	−0.260	7.9E-05****	0.98
*g01130*	6.272	−0.245	0.00023***	0.87
*Xumn10*	6.407	−0.398	9.3E-07****	1.42
*Xsnp3BS*-*9*	6.395	−0.366	1E-05****	1.26

^a^Where b0 = the y intercept, b1 = the slope and pr(F) = the p value associated with the F statistic

^b^The R^2^ (%) is the amount of phenotypic variation associated with the QTL

Significance at the 0.1 and 0.01 % levels are indicated by *** and ****, respectively

**Table 4:** QTL analysis performed for *Stagonospora nodorum* blotch resistance by interval mapping of F_2_ progeny derived from a cross of Arina (*QSng.sfr*-*3BS*) by an F_2_ progeny from a cross between Alsen and Ocoroni86 that was homozygous for the molecular markers linked to the *Fhb1* (Alsen) and *Sr2* (Ocoroni86) resistance genes

Marker	R^2^ (%)^a^	Additive effect^b^	Dominance effect^b^
*Xsnp3BS*-*2*	1.45	−0.39	−0.36
*Xsnp3BS*-*3*	0.99	−0.26	−0.26
*g01130*	1.62	−0.37	−0.39
*Xumn10*	1.85	−0.44	−0.39

^a^The R^2^ (%) is the amount of phenotypic variation associated with the QTL

^b^Additive and dominance genetic effects for an allelic substitution were estimated using QTL Cartographer 2.5. Negative numbers indicate that the effect came from Arina

## Discussion

The main objectives of this analysis were to determine the order of resistance genes *Fhb1*, *Sr2* and *QSng.sfr*-*3BS* on wheat chromosome arm 3BS and to move all three genes into a common genetic background. Analyses using shared markers on previously published linkage maps with MergeMap suggested that the SR resistance gene (*Sr2*) is the most distal, the FHB resistance gene (*Fhb1*) is the most proximal and the *P. nodorum* resistance gene (*QSng.sfr*-*3BS*) is between *Sr2* and *Fhb1* on the short arm of chromosome 3B of wheat. This hypothesis was supported by analysis of the 1600 F_2_ progeny that segregated for markers linked to all three resistance genes. Comparison of our linkage map and a recently published map of the *Fhb1*-*Sr2* region of chromosome 3BS based on a Ning7840/Clark BC_7_F_7_ population [40] supported this map order (Fig. 4). The Bernardo et al. map in 2012 [40] has loci *Xsnp3BS*-*2*, *Xsnp3BS*-*3*, *Xumn10*, and *Xsnp3BS*-*9* in common with ours and all were in the same order with similar distances in both maps.

The long-term goal of this research is to combine all three genes in coupling to develop a linkage block on 3BS with resistance to different fungal pathogens. This is of particular interest because *Fhb1* is the most commonly deployed gene for resistance against *F. graminearum*, *Sr2* is effective against SR race TTKSK (Ug99), and SNB is common in most wheat-growing areas worldwide. The gene order was not known when the project was initiated, and the most successful cross among the original three parents was between the spring wheat cultivars Alsen and Ocoroni86, having the most distal (*Fhb1*) and most proximal (*Sr2*) genes, respectively. From a population of 400 F_2_ individuals, we were able to recover two plants homozygous for marker alleles associated with both resistance genes, indicating that recombination had occurred in both gametes for each plant. The relative ease with which plants homozygous for *Fhb1* and *Sr2* were recovered allowed for rapid generation of a population segregating for all three loci. However, this then required development of a very large population when generating the final genetic linkage map utilizing genotypic data from F_2_ progeny from a cross with *Fhb1* and *Sr2* in coupling and *QSng.sfr*-*3BS* in repulsion.

Previously identified molecular markers that are tightly linked to *Sr2* and *Fhb1* segregating in the large F_2_ population were used as proxies for these resistance genes, and phenotyping was done with SNB only to avoid any confounding effects that might have arisen if all three diseases had been tested on the same plants. The histogram of SNB phenotypes was skewed towards susceptibility, which might reflect the assay conditions as well as inheritance of disease resistance. To ensure adequate infection, spikes were inoculated with freshly prepared inoculum and the spikes were covered for 3 days. These highly permissive conditions may have increased the severity of the disease and caused heterozygotes to have a susceptible phenotype. Unfortunately, the SNP locus (*g1130*) that was most closely linked to *QSng.sfr*-*3BS* behaved as a dominant marker in the cross so it was not possible to distinguish the large number of heterozygotes from either homozygote. This precluded an analysis to test how well the marker predicted the phenotype by itself.

It was difficult to determine the exact position of the *QSng.sfr*-*3BS* gene in the QTL analysis due to the small number of segregating loci, which spanned a narrow genetic window of only 10 cM. Single-marker analysis showed that all five SNP markers were highly significant, indicating the presence of a SNB resistance QTL in this region of 3BS. A highly significant QTL peak (Fig. 5) was identified approximately 1 cM proximal to the predictive SNP marker *g01130* for SNB. This locus was most closely associated with *QSng.sfr*-*3BS*, which was also supported by Shatalina et al. in 2013 [55]. Markers *Xsnp3BS*-*2* for *Sr2* and *Xumn10* for *Fhb1* were placed on either side of locus *g01130*. Therefore, it seems most likely that the first peak on the interval mapping analysis corresponds with *QSng.sfr*-*3BS*, approximately 1 cM proximal to *g01130* and about 4 cM distal to the marker for *Fhb1*. Inclusion of additional markers proximal to *Xsnp3BS*-*9* that are not associated with the resistance phenotype would anchor the QTL analysis and provide better definition to the peak; the wide peak with all markers significantly associated with resistance in Fig. 5 is most likely an artifact of an overly narrow genetic window. As expected for these distances, recombinant plants that appeared to have both *Sr2* and *QSng.sfr*-*3BS* were recovered at a lower frequency than those with *Fhb1* and *QSng.sfr*-*3BS* in coupling. In total, five recombinant plants with *QSng.sfr*-*3BS* in coupling with either *Fhb1* or *Sr2* were identified in the F_2_ population. Those recombinant progeny with two resistance genes in coupling can be used in further crosses to obtain recombinants having all three resistance genes in a single linkage block.

The low level of phenotypic variation explained by marker locus *g01130* as shown in Tables 3 and 4 could be due to the low number of polymorphic markers and quality of phenotypic data collected. As the study was conducted on an F_2_ population, phenotypic data were taken from single plants. Lack of replication increases error variance [56], which might have decreased the overall phenotypic variation (Tables 3 and 4). This could be addressed by increasing the marker density to increase the power to detect linked QTL [57], and by phenotyping F_2:3_ populations so that multiple plants can be phenotyped per entry. In addition, a recent study by Shatalina et al. in 2013 [58] suggested the presence of two genetically distinct SNB resistance loci in the *QSng.sfr*-*3BS* target interval. Marker loci *sun2* and *g01130* were tightly linked and were located between these two loci in the telomeric region of chromosome 3BS [55, 58]. After comparing our genetic map and two of the genetic maps from Shatalina et al. in 2013 [55, 58], SNB resistance locus *QSng.sfr*-*3BS* from our study was located distal to marker locus *sun2*, consistent with gene A in Shatalina et al. in 2013 [58]. These results indicate a high genetic complexity to the SNB resistance controlled by *QSng.sfr*-*3BS*. Presence of two linked resistance loci also could be the reason behind the wide, highly significant QTL (Fig. 5). This hypothesis could be tested by adding more markers and by scoring F_3_ families for resistance phenotype.

## Conclusions

Knowing the positional order of these resistance genes will enable the development of a wheat line with all three genes in coupling to provide durable and broad-spectrum resistance against multiple major diseases of wheat. The tight linkage between *Sr2*, *QSng.sfr*-*3BS*, and *Fhb1* shown from this study suggests that, once obtained, it should be relatively easy to maintain this linkage block in a breeding program. Previously published linkage maps have reported that leaf rust resistance gene *Sv2* is in the same general region and likely occurs between *Fhb1* and *QSng.sfr*-*3BS* (Fig. 2). Yellow rust (stripe rust) resistance gene *Yrns*-*B1* was reported on chromosome arm 3BS [42], proximal to the other genes (Fig. 2). Therefore, it may be possible in the future to combine *Sv2* and *Yrns*-*B1* with the other three genes in coupling for a linkage block with resistance against five important fungal pathogens of wheat.
